# Disease in Milk

**Published:** 1888-06

**Authors:** 


### DISEASE IN MILK.

The subject of purity and healthfulness of milk and its products has received much attention from medical and sanitary authorities during the past year, aud some very remarkable results of investigations are now being made public. A lecture on the etiology of scarlet fever was recently delivered by Dr. E. Klein, F. C. S., before the Royal institution in London. The principal theme of the paper was the relation of scarlet fever to milk supply. The possibility of the dissemination, and even origin, of the disease from this source was considered at length. Recorded cases are quoted to prove its possibility. The lecturer treats it as a certainty that milk has thus caused the spread of scarlet fever. Experiments by N. Galthier, a French scientist, have been published. These were directed to tubercular sickness. Dairy produce from cows affected with tubercular disease was the subject of the investigations. Professor Galthier found that such articles of diet could communicate phthisis or consumption to poultry and swine, and could become thus directly or indirectly a serious menace to man.

Within the last few years a number of outbreaks of disease have been traced with great certainty to dairies as the center of contagion. So well proved have these cases seemed, that they have originated special popular names for the sicknesses thus occasioned. Thus milk typhoid, milk scarlatina, and milk diphtheria have come to be recognized. In a number of accurately recorded cases, an outbreak of some specific disease has been noted. The general history in all was identical The spread was limited to a certain number of families. The medical officers found that all the families thus affected were supplied with milk from the same dealer. Then, on examining the stables or dairy whence the milk came, the source of contagion was manifest. A case of scarlet fever would be found in the family or among the employees, or some of the residents possibly had diphtheria. In a number of instances such conditions were established. At the present time the English health authorities consider these cases proved. They form the basis for a somewhat disquieting suspicion affecting our milk supply.

But their is a more alarming aspect of the question. The result of some of the more recent observations is that cows may themselves become infected with a sickness resembling scarlet fever, and that such cows may, by their milk, cause the true scarlet fever to be developed in human beings.

This conclusion has been led to by an examination of data in recorded cases. In some instances where the origin of the sickness was traced to milk, and where also a scarlet fever case had existed in some person connected with the dairy, too long a period elapsed before the breaking out out of the epidemic to allow it to be attributed to direct conveyance by the milk. Another class of cases is cited in which a human origin, proximate or ultimate, could in no way be traced. In one such instance an outbreak of scarlet fever was associated with a certain dairy. No human being could in any way be fixed upon as the originator. Even the sanitary conditions were examined, with negative results The disease was finally attributed to certain cows. Examination of them showed the presence of disease, whose symptoms included sores upon the body, ulcerations and a visceral complaint resembling that occurring in scarlet fever in the human being. The outbreak had, from other data, been limited to these cows as a source. Their disease so similar to the human scarlet fever made it almost a certainty that they were the origin of the trouble.

The examination by bacterial analysis was entered into and confirmed these suspicions. The same micrococcus was found in the blood of scarlet fever patients and in the affected cows. The action of the human microbe on animals was identical with that of the vaccine one. This investigation, a full outline of which it is needless to give, clinched the proof. Succeeding occurrences investigated in the same general way gave identical results.

It may be considered as clearly proved that milk can be a serious source of danger to health or life. The remedy is a simple one. By heat the micrococci are destroyed. If the milk is heated to 285 deg. F., it will be rendered safe. Any infections microbes present will be killed. But while this disposes of the milk it does not touch the disposal of milk products. Butter, cream and cheese are all uncooked. Butter represents raw fat, or uncooked oleaginous matter. It cannot be heated to a high degree without injury. One of the methods of freeing it from casein was to melt it, but the process was found to cause deterioration. Butter must be uncooked.—*Scientific American.*

---

In the healing of burns and scalds, where there is danger of contracting scars, rub the new skin several times a day with good sweet oil. Persist in this rubbing until the skin is soft and flexible.

